# Impact of physical activity in vascular cognitive impairment (AFIVASC): study protocol for a randomised controlled trial

**DOI:** 10.1186/s13063-019-3174-1

**Published:** 2019-02-11

**Authors:** Ana Verdelho, Sofia Madureira, Manuel Correia, José Manuel Ferro, Mário Rodrigues, Manuel Gonçalves-Pereira, Mafalda Gonçalves, Ana Catarina Santos, Pedro Vilela, Helena Bárrios, Mariana Borges, Helena Santa-Clara

**Affiliations:** 1 Department of Neurosciences and Mental Health, Faculdade de Medicina, Centro Hospitalar Universitário Lisboa Norte Hospital de Santa Maria, Instituto de Medicina Molecular (IMM) and Instituto de Saúde Ambiental (ISAMB), Universidade de Lisboa, Avenida Professor Egas Moniz, 1649035 Lisbon, Portugal; 2 Department of Psychology, ISCTE-IUL, NOVA Medical School / Faculdade de Ciências Médicas, Universidade Nova de Lisboa, Instituto de Medicina Molecular (IMM), Faculdade de Medicina, Universidade de Lisboa, Lisbon, Portugal; 3 Neurology Service, Hospital de Santo António, Centro Hospitalar do Porto and Instituto de Ciências Biomédicas Abel Salazar (ICBAS), University of Porto, Porto, Portugal; 4 Instituto de Medicina Molecular (IMM), Faculdade de Medicina, Universidade de Lisboa and Department of Neuroscience, Hospital de Santa Maria-CHLN, Faculdade de Medicina, Universidade de Lisboa, Lisbon, Portugal; 5 Instituto de Medicina Molecular (IMM), Faculdade de Medicina, Universidade de Lisboa, Lisbon, Portugal; 6 CEDOC, Chronic Diseases Research Center, NOVA Medical School /Faculdade de Ciências Médicas, Universidade Nova de Lisboa, Lisbon, Portugal; 7 Instituto de Medicina Molecular (IMM), Faculdade de Medicina, Universidade de Lisboa, Lisbon, Portugal; 8 Instituto de Medicina Molecular (IMM), Faculdade de Medicina, Universidade de Lisboa and Dementia Unit, Hospital do Mar, Lisbon, Portugal; 9 Neuroradiology - Imaging Department, Hospital da Luz, Lisbon, Portugal; 10 NOVA Medical School, Universidade Nova de Lisboa, Portugal and Instituto de Medicina Molecular (IMM), Hospital do Mar Lisboa, Faculdade de Medicina, Universidade de Lisboa, Lisbon, Portugal; 11 Instituto de Medicina Molecular (IMM), Faculdade de Medicina, Universidade de Lisboa and Faculdade de Motricidade Humana,Universidade de Lisboa, Lisbon, Portugal; 12 Faculdade de Motricidade Humana, Universidade de Lisboa, CIPER – Centro Interdisciplinar de Estudo da Performance Humana, Lisbon, Portugal

**Keywords:** Vascular cognitive impairment; physical activity, Randomised, Stroke, TIA, Cerebral small vessel disease

## Abstract

**Background:**

Cognitive impairment and cerebrovascular pathology are both frequent with ageing. Cognitive impairment due to vascular pathology of the brain, termed vascular cognitive impairment (VCI), is one of the most frequent causes of cognitive impairment in elderly subjects. Thus far, VCI has no specific pharmacological treatment. Recent observational studies have suggested a protective effect of physical activity in cognition, but adequate randomised controlled trials (RCT) are lacking.

**Methods:**

AFIVASC is a multi-centre randomised controlled trial, with a 6-month intervention treatment and an additional follow-up of 6 months, that aims to estimate the impact of 6 months of moderate intensity physical activity on cognition (the primary outcome) at 6 and 12 months in subjects with VCI. Participants are community dwellers with criteria for VCI without dementia or who have had previous stroke or transient ischaemic attack (TIA). Patients may be self-referred or referred from a medical appointment. After confirming the inclusion criteria, a run-in period of 1 month is conducted to access adherence; only after that are subjects randomly assigned (using a computerised program blinded to clinical details) to two groups (intervention group and best practice usual care group). The intervention consists of three physical activity sessions of 60 min each (two supervised and one unsupervised) per week. The primary outcome is measured by the presence or absence of decline in cognitive status. Secondary outcomes include changes in neuro-cognitive measures, quality of life, and functional and motor status. Primary and secondary outcomes are evaluated at 6 and 12 months by investigators blinded to both intervention and randomisation. A required sample size of 280 subjects was estimated. Statistical analyses will include regression analysis with repeated measures. The study was approved by the Ethics Committee for Health of Centro Hospitalar de Lisboa Norte (ref. no. 1063/13) and by the Ethics Committee for Health of Centro Hospitalar do Porto CHP (ref. no. 2016.055(049-DEFI/048-CES)).

**Discussion:**

We aim to show whether or not moderate physical activity has a beneficial impact on cognition, quality of life, motor, and functional status in people with vascular cognitive impairment, and to generate new insights on the applicability of implementing physical activity in this specific population.

**Trial Registration:**

ClinicalTrials.gov, NCT03578614 July 6, 2018.

**Electronic supplementary material:**

The online version of this article (10.1186/s13063-019-3174-1) contains supplementary material, which is available to authorized users.

## Background

Cognitive impairment is frequently associated with ageing, and cerebral vascular pathology is one of its most frequent causes [1, 2]. Vascular cognitive impairment (VCI) includes a myriad of clinical conditions having cerebrovascular aetiology in common that ultimately leads to some degree of cognitive impairment. Cognitive impairment ranges from single domain impairment, behavioural changes and mild multi-domain impairments to dementia (the most severe stage, termed vascular dementia). Dementia is characterised by a compromise of several cognitive domains that leads to functional decline (either in the social, occupational or personal and family context) and loss in autonomy in performing activities of daily living of different complexity. In milder forms, cognitive impairment due to vascular aetiology can be difficult to diagnose since it may be expressed by symptoms which tend to be linked to ageing and not to memory impairment. Examples of these symptoms are reduced verbal or motor initiative, psychomotor slowness, difficulty in multi-tasking, difficulty in planning, sequencing and finalizing actions, or even behavioural manifestations only (shown as less interest in hobbies or usual activities, and irritability or distractibility). VCI is caused by a heterogeneous group of vessel disorders causing different types of vascular lesions in the brain, from subclinical small vessel disease (including white matter changes, lacunes, or microbleeds) to clinically overt stroke [1, 2].

There is no specific treatment for VCI, and pharmacological trials have thus far generated negative results [3, 4]. Therefore, in the absence of a cure, efforts should be made to improve research and intervention in vascular risk factors [4–6]. Physical activity has gained increasing interest as a non-pharmacological treatment for both primary prevention and for delaying evolution in cognitive impairments due to vascular disease. In animal studies, physical activity has been shown to produce several metabolic effects (increased expression of neurotransmitters, increased hippocampal cell proliferation and neurogenesis, increased antioxidant capacity, reduced inflammatory cytokines and oxidative stress, and improved mitochondrial functioning and vascular endothelial function), leading to better neuroplasticity [7]. In human functional and cerebral perfusion studies, physical activity was also associated with improvement in brain functioning [8].

The World Health Organization (WHO) (2010) [9] and the American College of Sports Medicine (2017) [10] have produced recommendations on physical activity. The associations of cardiovascular [11] and cerebrovascular diseases [12] also produced recommendations on physical activity for cardiac and for stroke patients, respectively. However, data on cognitive disorders are quite controversial. Recent observational studies suggested that physical activity could prevent the progression of VCI [13]. On the other hand, a recent review found no protective effect of physical activity in cognitive decline [14]. In the DAPA trial published recently, no beneficial effect was achieved with physical activity in patients with Alzheimer’s disease [15]. There have been intervention trials in patients at high vascular risk without previous stroke but, with the exception of one study [16] (which did not include VCI), no benefit was found [17, 18].

Given these contradictory findings, we lack a robust evidence base to recommend physical activity in VCI. Furthermore, there are no data on what type, intensity, and frequency of activity would be necessary to achieve long-term gains.

We aim to test, through a randomised controlled trial (RCT), if physical activity has a beneficial impact on VCI. We hypothesise that regular physical activity of moderate intensity for at least 6 months will improve cognitive impairment in subjects with cognitive impairment due to cerebrovascular pathology.

We also aim to evaluate the effect of physical activity on the quality of life, and on functional and motor status.

## Objectives

The primary objective is to conduct an RCT to evaluate the effect of 6 months of physical activity on cognitive performance in subjects with VCI without criteria of dementia at inclusion. We aim to evaluate cognitive performance (as the primary outcome), and quality of life and motor performance and functional status (as secondary outcomes). Other objectives are to identify the determinants for the progression of cognitive decline and quality of life in a large Portuguese sample of subjects with VCI without dementia and also to evaluate the impact of the engagement in physical activities in both subjects and informants.

## Methods/design

### Design

**Fig. 1:**
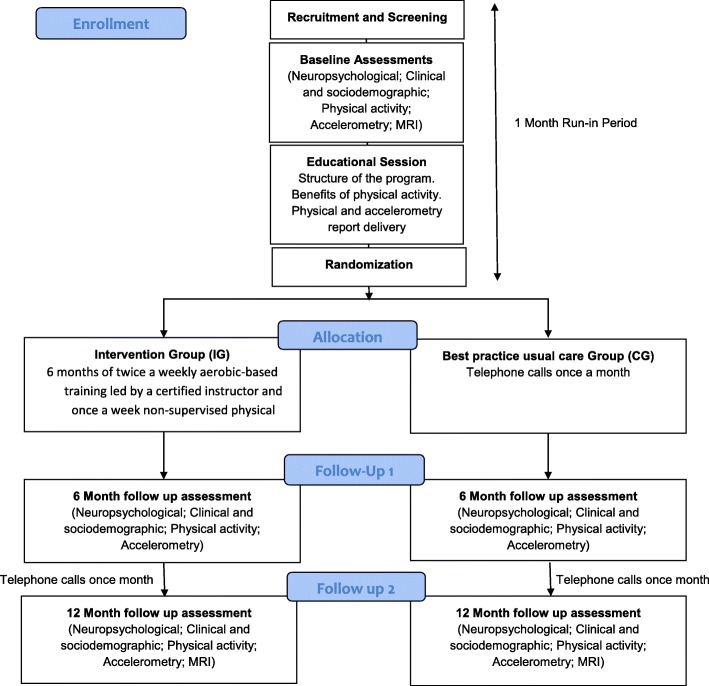
Study design. MRI magnetic resonance imaging

AFIVASC (“physical activity in vascular cognitive impairment’) is a Portuguese multi-centre, randomised parallel group trial comparing a 6-month intervention of two moderate physical activity sessions and one unsupervised moderate physical activity session per week with an additional follow-up of 6 months to evaluate its long-term impact in comparison with the current best practice usual care. The Consolidated Standards of Reporting Trials (CONSORT) statement [19] has been used as a framework for the development of our methodology (Fig. 1). To ensure adherence, a run-in period will be conducted after inclusion. This run-in period consists of a 4-week period before randomisation during which all participants are assigned to equal procedures, namely the educational session and all planned assessments. After the run-in period, participants are randomised into two parallel groups (best practice usual care group and the intervention group). The best practice usual care group will receive the standard usual care, but they are allowed to perform physical activity by self-initiative, although supervised physical intervention is not allowed. The control of the amount of physical activity per week is performed in both groups through accelerometry. We do not expect to see high levels of physical activity in the usual care group since VCI is usually associated with a reduced initiative.

All participants will be followed up for 12 months. Informants (usually family members) are recruited, as is usual in cognitive studies, to provide additional data since we expect the sample size to decline over time. Participants, informants, and exercise physiologists are not blinded to the treatment, but all baseline and follow-up assessments (physical, neuropsychological, and radiological) are blinded to treatment allocation. According to data protection rules, all data are anonymised. Technicians involved in statistical analysis will be blinded to treatment allocation.

### Participants

#### Recruitment and study setting

The study is conducted in the University of Lisbon at the Faculdade de Medicina and Instituto de Medicina Molecular in Lisbon, and the Hospital de Santo António, Centro Hospitalar do Porto. The Human Kinetics Faculty (from the University of Lisbon) is a partner of the study. These centres, both urban and academic, have experience in the diagnosis and management of patients with cognitive decline of a vascular aetiology and in stroke care. Subjects are referred to each participating centre directly from the community (participants can be self-presenting, as the study was advertised in public spaces) or from a medical appointment (general family doctor in primary care, or from neurological, mental health, memory clinics, or hospital settings). Participants can be referred to the study due to minor cognitive complaints/symptoms or minor behavioural changes attributed to a cerebrovascular aetiology. Patients can also be referred if they had a previous stroke or transient ischemic attack (TIA), even without complaints, if neuropsychological evaluation showed evidence of any change considered to be due to a cerebrovascular aetiology. Post-stroke cognitive impairment is common even after successful clinical recovery from a stroke episode, and a recent study showed that at least one cognitive domain was impaired in 83% of cases [20]. Considering TIA, more than one cognitive domain was impaired in more than a third of patients 3 months after a TIA [21].

Eligible subjects are contacted by the recruitment team to further explain the procedures of the study and to confirm inclusion and exclusion criteria. Written informed consent is given by participants and informants. This consent allows access to the subject’s clinical records for research purposes and indicates their willingness to participate in all the activities inherent to the study. Those remaining eligible and without contraindications to physical activity are contacted by an investigator and proceed to baseline assessments.

### Inclusion and exclusion criteria

#### Inclusion criteria

Participants are included if they are 18 years and older and fulfil the following criteria: 1) provide written informed consent; 2) are fluent in the Portuguese language; 3) are able to read and write; 4) have an available, reliable informant; and 5) show clinical and functional criteria A and B as below.

Criteria A (any one of the following three):

1. Probable mild cognitive vascular impairment (VCI, no dementia) [22];
2. Previous ischaemic or haemorrhagic stroke (more than 6 months before), with modified Rankin score ≤ 2 at baseline and without formal indication for physiotherapy;
3. TIA (more than 1 month before), diagnosed by a neurologist or with an identified vascular lesion (correlated with TIA clinical symptoms) in cerebral computed tomography (CT)/magnetic resonance imaging (MRI).

Criteria B: No functional changes, i.e. instrumental activity of daily living (IADL) scale = 0 (no item changed, or one single item with minimal change), according to the scoring methods of the LADIS study (minimum of four items applicable) [23] or no cognitive changes regarding the suggested Montreal Cognitive Assessment Test (MoCA) cut-off point for dementia in Portuguese clinical samples (score < 17) [24].

#### Exclusion criteria

Subjects cannot be included if they have at least one of the following: 1) a diagnosis of dementia; 2) stroke with formal indication for physiotherapy or speech therapy, or Rankin score ≥ 2; 3) any contraindication for walking, physical limitation to gait (orthopaedic or of other structural causes) that compromises physical activity, or other condition potentially interfering with the active treatment (e.g. severe arthritis or severe musculoskeletal pain associated with walking); 4) evidence of neurodegenerative disease (of aetiology other than vascular), severe mental disorder (e.g. major depressive episode or psychosis) or medical disease which could significantly interfere with the subjects’ participation or with their quality of life (e.g. cancer, congestive heart failure, or unstable angina).

### Study outcomes

The primary outcome measure will be cognitive decline defined as a dichotomous variable (decline/no decline) through cognitive status criteria as described in the Assessments section below. The secondary outcomes are changes in neurocognitive scores of at least 1.5 standard deviation (SD) from baseline to endpoint (using *z* scores), a decline in the raw values of neuropsychological tests, in quality of life, functional and motor status, and falls.

### Assessments

**Fig. 2:**
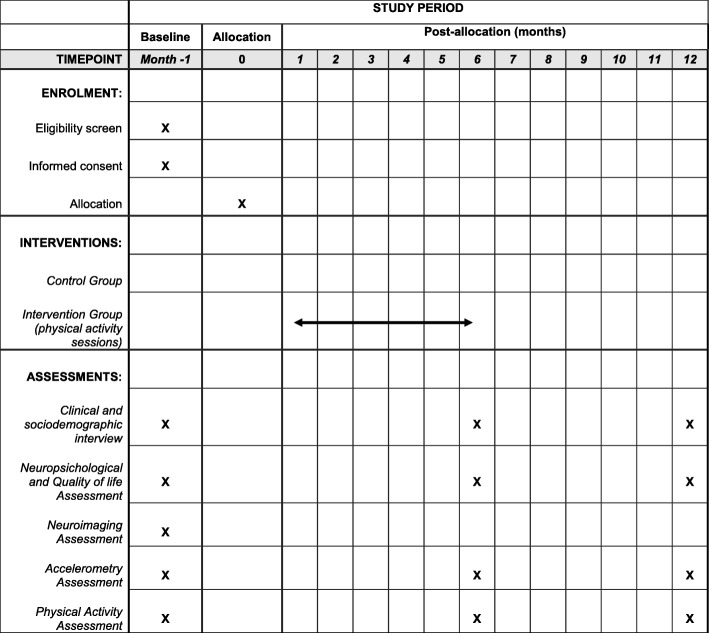
Study schedule

All participants attend three visits (baseline and two follow-up) consisting of clinical neuropsychological, physical, and functional observations (baseline, 6 months, and 12 months). The study schedule is shown in Fig. 2. In these visits, participants undergo the following assessments: 1) clinical and sociodemographic assessment; 2) physical activity assessment; 3) accelerometry assessment; 4) neuropsychological assessment; 5) and brain imaging (MRI scan; this only at baseline). Informants (appointed by participants as the best available person to provide information about them) are also interviewed.

In all assessments (baseline and follow-up clinical visits), the cognitive status of patients is classified into the following groups: 1) diagnosis of dementia; and 2) diagnosis of cognitive impairment with no dementia according to clinical, neuropsychological, and functional criteria. For this purpose, we used the usual criteria and definitions of the Diagnostic and Statistical Manual of Mental Disorders (edition 4 revised; DSM-4TR) [25]. At baseline, patients could only fulfil VCI without dementia defined as evidence of cognitive impairment and clinical consensus to identify significantly related vascular features; exclusion of dementia when impairments were not sufficiently severe to interfere with social or occupational functioning or when impairments were more focal than the global requirement for a diagnosis of dementia. For follow-up we considered the following criteria for subtypes of dementia: probable Alzheimer disease [26]; probable vascular dementia [27]; subtype of subcortical vascular dementia [28]; frontotemporal dementia [29]; and Lewy body dementia [30]. The criteria for Alzheimer’s disease with a vascular component was made when the investigator judged that the clinical picture presented both aspects of Alzheimer’s disease and vascular dementia.

### Assessment measures

Participants are interviewed regarding sociodemographic factors relevant to the study subject and clinical factors, including cognitive status in a neurological assessment and functional status evaluation with the modified Rankin scale and the National Institutes of Health Stroke Scale (NIHSS) [31–33].

### Neuropsychological and quality of life assessments

Cognitive evaluation follows the same protocol in all assessments and is applied by a trained neuropsychologist. It consists of a neuropsychological battery specifically designed for this study in order to be sensitive for vascular cognitive deficits and includes the following tests: The Montreal Cognitive Assessment (MoCA) [34, 35] for general assessment; Letter cancellation [36] and the Wechsler Adult Intelligence Scale-Revised (WAIS-R) Digit Span (forward) [37, 38] for the assessment of attention and concentration; Verbal Fluency [35], Trail-Making Test [39–41], Wechsler Adult Intelligence Scale-3rd edition (WAIS-III), Digit-Symbol [42], and Stroop Test [43, 44] to assess executive functions; the California Verbal Learning Test-9 (CVLT-9) [45, 46], Wechsler Memory Scale (WMS) Visual Reproduction and Logical Memory subtests [36, 47], and WAIS-R Digit Span (backward) subtest [36, 37] to assess memory; and the Alzheimer's Disease Assessment Scale (ADAS) Naming objects and Following Commands subtests [48, 49] to assess language. The impact of cognitive performance on daily living activities is assessed with the Instrumental Activities of Daily Living (IADL) [50]; the presence of depressive symptoms and apathy are assessed using the 15-item Geriatric Depression Scale [51] and the Apathy Evaluation Scale [52, 53], respectively.

The quality of life and other specific measures for the assessment of an individual psychological resources and the economic impact of the disease are also added in this protocol. These measures include the Euro Qol-5 [54] and the Quality of Life in Alzheimer’s Disease (QOL-AD) [55, 56], the 13-item Orientation to Life Questionnaire to measure Antonovsky’s sense of coherence (SOC-13), a health-promoting resource which strengthens resilience where higher scores indicate an ability to adapt to stressful situations [57–59], the Successful Aging Index (SAI) [60], an a priori index model of successful ageing which was validated with respect to service use, and the Resources used in Dementia Interview (RUD) [61] to collect data on resource utilisation to calculate costs of care (healthcare resource utilisation) and time dedicated to caregiving in dementia.

### Physical activity assessment

The Functional Physical Fitness Battery [62] is a simple, reproducible, readily available tool to assess submaximal functional capacity and to evaluate the response to intervention. The Six-Minute Walk Test (6MWT) is performed around an 18-m line to measure the distance in metres walked in 6 min. Participants are instructed to walk at their own pace according to their tolerance to exercise for 6 min, with rest stops as needed.

The 30-s chair stand assesses lower body strength, which is needed for numerous tasks such as climbing stairs or walking. Participants are instructed to sit and stand as fast as they can in 30 s with arms folded across the chest.

To assess lower body flexibility (which is important for good posture, normal gait patterns, and for various mobility tasks, such as getting in and out of a bathtub or car), the participant is asked to start from a sitting position at the front of the chair, with one leg extended and hands reaching toward the toes. The distance in centimetres between the extended fingers and the tip of the toe are measured in both legs. To assess upper body (shoulder) flexibility (which is important in tasks such as combing one’s hair, putting on overhead garments, and reaching for a seat belt), the participant is asked to reach over the shoulder with one hand and up the middle of the back with the other hand. The distance in centimetres between the extended middle fingers is measured.

The agility/dynamic balance is assessed by the number of seconds required to get up from a seated position, walk 2.44 m, turn, and return to the seated position. In this framework, it is interesting to measure multiple dimensions of balance in different sensory environments; therefore, the Fullerton Advanced Balance Scale (FAB) is also applied [63]. The FAB comprises 10 items that are scored using a 0–4 ordinal scale (only eight items are used due to safety and functional reasons). In this case, the highest score possible on the test is 32 points. Items include standing with the feet together and eyes closed, reaching forward to retrieve an object (a pencil) held at shoulder height with an outstretched arm, turn 360 degrees in both right and left directions, a tandem walk, standing on one leg, standing on foam with the eyes closed, a two-footed jump, and a walk with head turns.

### Accelerometry assessment

The physical activity (PA) time of each participant is assessed by accelerometry (ActiGraph, GT3X+ model). The accelerometer is a device that measures the acceleration of normal human movements, ignoring high-frequency vibrations associated with mechanical equipment. All participants are asked to wear the accelerometer on the right hip, close to the iliac crest, for a week. The device activation, download, and processing is performed using Actilife v.6.13.3 software (ActiGraph, Fort Walton Beach, FL, USA). The devices are activated on the first day before the interview and data are recorded using the raw mode with a 30-Hz frequency and posteriorly downloaded into 15-s epochs.

A valid day is defined as having 600 min (10 h) or more of monitor wear and all participants with at least 3 valid days (including 1 weekend day) are included in the analyses. Accelerometer counts ≥ 100 counts per minute (cpm) are classified as PA with additional separation into light intensity (100–2019 cpm) and moderate-to-vigorous intensity (≥ 2020 cpm) [64, 65]. There are no cut-offs for the sedentary time using the three-axial information from this new generation Actigraph GT3X+ accelerometer; therefore, we used the previous cut-offs which are based on the vertical axis only. Compliance with PA recommendations for public health is assessed according to the WHO recommendations (adults: 150 min/week of at least moderate-to-vigorous intensity defined as ≥ 21.4 min/day).

### Neuroimaging assessment

Besides confirming eligibility for the study, MRI is further used to control for confounders (for instance, information on the degree of atrophy, severity, number and localisation of vascular lesions, and presence of strategic lesions). MRI uses the following sequences: Fast Spin Echo, Fast Flair, T1 W-3EDGE (MPRAGE), diffusion and SWI, using a previously defined protocol based on the LADIS European study [66]. The parameters that are measured include:

1. Atrophy (Scheltens evaluation, score 1–8 for global atrophy and 0–4 for temporal lobe atrophy) [67];
2. White matter change evaluation using Modified Fazekas scale for severity (score 1–3) [68] and Scheltens scale for localisation of the lesions [67];
3. Number and localisation of microbleeds (SWI sequences);
4. Number and localisation of lacunes;
5. Description of previous infarcts and intracerebral haemorrhages and silent infarcts.

MRI is conducted on the total sample at baseline. In all centres the MRI machine will have the same power (3 T).

To check for adhesion to the MRI protocol and the quality of scans, the performance of a dummy run will be required before the start of the study. For validation, the representative images of the dummy run will be checked centrally before start inclusion, and corrections will be made if necessary.

MRI will have a central reading, blinded to the clinical details, conducted by the responsible neuroradiologist. A clinical neurologist (JMF) not involved in the clinical assessment will also review the images blinded to the clinical results.

### Run-in period and follow up assessments

The use of a pre-randomisation run-in period aims to ensure the engagement and adherence of subjects and to reduce bias associated with different levels of knowledge concerning expected benefits of the intervention. Over 4 weeks there is an educational group session, where the structure of the study, the benefits of physical activity, and the accelerometry report are explained.

Follow-up assessments will be conducted by clinicians (neurologist, neuropsychologist, and exercise physiologist not involved in the intervention sessions and blinded to the arm).

### Randomisation and masking

After the 4-week run-in period, research personnel not involved in the recruitment, assessment, or intervention are responsible for managing the randomisation process using a statistical program. The randomisation sequence is generated by R software [69]. Participants are randomly assigned (1:1) to either the intervention group (IG) or to the best practice usual care group (CG), stratified by gender.

All follow-up assessments (physical, neuropsychological, and clinical diagnosis), and also the neuroradiological evaluation, are blinded to treatment allocation, as well as in the data set registry.

### Intervention

#### Usual care

All participants receive usual care according to national guidelines. The Direção Geral da Saúde (DGS) only has guidelines for the elderly and not for subjects with VCI. In these guidelines, physical activity is recommended without further specification [70]. When clinically relevant problems are identified, the doctor in charge of the patient will be contacted for subsequent guidance.

#### Experimental intervention and implementation

In the intervention group, three physical activity sessions are planned per week (two supervised and one unsupervised) conducted over 6 months. In the first 2 months, the supervised sessions have 10 min of warm-up plus 5 min active pause (balance, agility, and coordinative exercises) plus 15 min walking plus 5 min active pause (resistance exercises, 1 series of 12 repetitions, three callisthenic exercises) plus 15 min walking plus 5 min flexibility (1 series of 10 s in three different postures). The aimed intensity in these first 2 months is 12/13. Between the second and fourth months, the duration of the walking period increases, as well as the intensity (rating of perceived exertion (RPE) 13/14). The session consists of 10 min of warm-up plus 5 min active pause (balance, agility, and coordinative exercises) plus 20 min walking plus 5 min active pause (resistance exercises, 1 series of 15 repetitions, three callisthenic exercises) plus 15 min walking plus 5 min flexibility (1 series of 10 s in three different postures). Between months 4 and 6, the duration of the walking period again increases, as well as the intensity (RPE 14/15). The session consists of 10 min of warm-up plus 5 min active pause (balance, agility, and coordinative exercises) plus 25 min walking plus 5 min of aerobic functional exercises (1 series of 15 repetitions, three callisthenic dynamic exercises) plus 20 min walking plus 5 min flexibility (1 series of 10 s in three different postures).

To measure the intensity level of the physical activity, the Borg rating of perceived exertion (RPE) ranging from 6 (rest) to 20 (maximum effort) is used [71]. Perceived exertion is how hard you feel your body is working. It is based on the physical sensations a person experiences during physical activity, including increased heart rate, increased respiration or breathing rate, increased sweating, and muscle fatigue. Although this is a subjective measure, a person’s exertion rating may provide a fairly good estimate of the actual heart rate during physical activity [71].

For the unsupervised session, participants are asked to accumulate at least 3 bouts of 10 min walking during the day, with the RPE according to their training intensity phase.

Regarding the best practise usual care group, physical activity counselling is given during the educational session before the randomisation based on their accelerometry results.

#### Intervention attendance and adherence

Intervention attendance within the 6 months is recorded in log sheets at the beginning of each session by the exercise physiologist responsible for the supervised physical activity sessions. For the unsupervised physical activity session, patients are asked in the second session of each week to report whether they have performed it or not. Dropouts are registered and, whenever the subjects allow, follow-up is maintained with the same design, although the subject cancels participation in the sessions.

### Data management and safety

Data management follows the General Data Protection Regulation and the National Clinical Trial regulation (Act n° 21/2014, 16 April). Case report forms (CRFs) only include an anonymised code. Personal identification or any data leading to personal identification is not directly associated with data collection forms. The personal identification of subjects and data collection forms are stored in different locations of the leading centre, and data is only accessed by the research team.

Adverse events are registered in all scheduled appointments and reviewed by the exercise physiologist in all sessions. Adverse events are registered in the CRF. Serious adverse events are defined in the protocol as adverse events that require hospitalisation, medical interventions, or implicate significant disability, death, or are immediately life-threatening. These implicate referral to the doctor in charge, or to the emergency room if applicable. We should note that we do not expect to have relevant serious adverse effects, as the intervention follow WHO guidelines.

### Study supervision and ethics

The Steering Committee includes the principal investigator (AV), the investigator leader in the second centre (MC), the leader in human kinetics (HSC), and the neuropsychologist expert leader (SM). Two independent members make up the data monitoring team (one an expert in statistics (ACS) and the other a clinician (HB)). Any protocol modifications are required to be submitted to the Ethical Committee after discussion within the Steering Committee. Dissemination of study results is planned both in scientific and in public sessions.

All procedures of the study and the CRFs, as well as the consent form, were approved by the Ethics Committee for Health of the Centro Hospitalar de Lisboa Norte (ref. no. 1063/13) and by the Ethics Committee for the Health of Centro Hospitalar do Porto CHP (ref. no. 2016.055(049-DEFI/048-CES)). As the intervention follows the WHO guidelines, the Ethical Committee did not consider it necessary to provide compensation in case of any incident during the study.

### Statistics

#### Sample size

The sample size calculation is based on two sets of data. In the LADIS observational prospective study, which included a similar population [13], there was progression in cognitive decline in 31% of the initial sample after 1 year of follow-up (200 participants out of 639 participants included). In the Osaki-Tajiri Project [72], which studied conversion to dementia in a similar population and measured different severities of cerebrovascular pathology (as in our study), odds ratios (ORs) between 2.12 and 6 were found to be implicated in the conversion into vascular dementia. We used an OR of 4 in our calculation of the expected effect (which is clinically significant).

Using Open Epi software [73] for sample calculation in randomised trials, we realised a number of 117 subjects in each arm with 95% confidence intervals (alfa of 0.05) and 80% power, giving a total of 234 subjects to be recruited. Loss to follow-up is usually 20% in this type of follow-up study, and thus a total of 280 subjects is estimated as being needed.

#### Data analysis

Data will be registered according to the Consolidated Standards of Reporting Trials (CONSORT) guidelines for randomised controlled trials [19]. We will use intention-to-treat analyses as the primary analysis with all participants included according to initial group allocation. For the primary outcome analysis (dependent variable: cognitive decline according to clinical criteria), we will conduct a logistic regression analysis. Since the outcomes are measured at 6 and 12 months, we will fit a generalised estimating equations (GEE) logistic regression model for the data. The models will adjust for covariates which are assumed to interfere with cognition in the follow-up (age, gender, education, and baseline MoCA). For secondary outcomes, logistic or linear regression analysis will be used as applicable.

For each of the outcome endpoints (i.e. transition of cognitive status and change in neuropsychological evaluation through composite or global scores) the change from baseline to 6 months and 12 months will be assessed, even though we will consider 12 months as the primary marker of success.

Observing a statistically significant difference on any of the outcomes will be considered preliminary evidence of efficacy. We will also report variances, co-variances, and effect sizes, as well as sampling feasibility (i.e. ease of recruitment, recruitment rate, withdrawal rate).

The same qualified statistician involved in the statistical analysis designed the SAP and will independently review the analysis according to the plan.

## Discussion

There is considerable controversy concerning the impact of physical activity on the prevention of cognitive impairment of vascular aetiology. Most of the existing evidence is garnered from observational studies, with considerable heterogeneity of methods and types of interventions. Physical activity is currently recommended for the prevention of cardiovascular diseases and is already included in the European Guidelines of Cardiovascular Prevention and Rehabilitation [11], but evidence in neurocognitive disorders is lacking. To the best of our knowledge, there are no previously published, high-quality parallel control trials on the effect of physical activity on the cognitive status of people with cognitive impairment due to cerebral vascular pathology. RCTs in other cognitive outcomes (for instance in Alzheimer’s disease) have generated controversial results [15]. In the recent DAPA trial [15], physical activity not only failed to improve cognitive status, but was associated with a mild (although not significant) cognitive deterioration. Regarding our study, we are aware of some limitations associated with the characteristics of any randomised controlled trial design, implying a selection of participants and exposure conditions that, in the end, may preclude generalisability. Nonetheless, in the absence of evidence-based interventions/treatments in vascular cognitive impairment, and considering that this is a highly prevalent pathology, we consider our study quite relevant. We believe that, in the field of neurocognitive disorders, vascular patients are more likely to benefit from physical interventions than patients with other neurodegenerative conditions. We also believe that if this study shows positive findings we will be able to deliver a simple and universal preventative treatment that can be widely disseminated in routine practice. At the same time, we are interested in determining the impact of physical activity on health-related quality of life and the motor and functional status of these patients, variables that have been understudied in this domain. Finally, we hope to explore the determinants of vascular cognitive impairment in Portuguese subjects.

## Additional file

Additional file 1: SPIRIT 2013 checklist: recommended items to address in a clinical trial protocol and related documents. (DOC 121 kb)
